# Remarks on the Constituents of Food and Drink

**Published:** 1855-01

**Authors:** Sanford B. Hunt


### ART. II.—*Remarks on the Constituents of Food and Drink.*

By Sanford B. Hunt, M. D.

### ART. I.—COMMON SALT, (CHLORIDE OF SODIUM.)

It is our purpose to present a series of articles on the physiological and therapeutical effects of those articles of food and drink which are in daily use. Many of these articles have decided chemical reäctions. Others possess in themselves medicinal powers which may often aid, or seriously interfere with, the action of remedies prescribed by the physician.

While it is doubtless true that a general rule for healthy diet can be assumed, which shall have a very wide adaptation to all sorts of people, yet it is very evident that the idiosycrasies or tastes of different individuals must often exert a wide influence upon the healthfulness of a common diet.

Individuals have their likes and dislikes. Some consume much fat, others but little, and the parallel may be carried out without limit. Every housekeeper is more or less puzzled to adapt her viands to the tastes of the various members of her family, and though a group may have been educated alike in matters of food, it will still be found that each one has a favorite dish, or a condiment peculiar to himself.

To ascertain what forms of food, drink, or condiment, are most suitable to special organizations is the object of these essays.

We commence with commom salt as an article which mixes more or less with the food of all nations, and all classes in society. First mentioned by Moses and Homer, it exists in all parts of the world, in almost all of the different geological formations, and most abundantly in the waters of the sea. All nations, and nearly all animals require it. The ruminantia, almost without an exception, manifest a fondness for it, and the salt licks of the west are marked by the footprints of many animal tribes.

Salt mingles in our food in a variety of ways. To many vegetables, to our bread, butter, and our fresh meats, it is added for the sake of its agreeable flavor, and in such uses it may be regarded as strictly a condiment. Its useful antiseptic powers also induce us to use it as a preservative of meats intended for long keeping. The salt pork and beef, and the ham of the farm house cellar, form no small part of the food of the people, and occasion a much larger consumption of it than would otherwise occur.

The constant presence of common salt in the blood and in some of the secretions, as in the bile, the urine, and the tears, is an evidence that it is not an adventitious component of the system, but rather a necessary and essential constituent thereof.

The recent experiments of M. Claude Bernard, upon the physiology of gaseous absorption, have thrown some light upon the office of salt as found in the blood. The conclusions which, in our present knowledge, may be drawn from its presence in the secretions, are too barren to be of much use. Always found in the tears, its quantity is so small that we can assign no general function to it there. A word of hypothesis may not, however, be out of place. In cholera patients there is not only a deficiency of saline ingredients in the blood, but also a deficiency of glandular secretion, so that the surface of the eye becomes unusually and sometimes entirely dry and unlubricated. Under these circumstances we have known several instances of sloughing of the cornea, by either desiccation, or ordinary gangrene. The nutrition of the cornea being carried on by the liquor sanguinis, we may draw the conclusion that it does not exist in these cases in sufficient quantity to maintain the life of the organ. But how far the absence of the antiseptic properties of the plasma, from the salt contained in it, may contribute to this result, must remain unsettled; except that the instances mentioned increase the probability that the office of the tears is not only lubricating, but antiseptic to the imperfectly nourished cornea.

We should infer similar properties in the bile, were it not that although the bile is strongly antiseptic, yet the pancreatic secretion, also containing some salt, is destructive in its action, and hastens putrefaction of the alimentary bolus when unchecked by the presence of bile.

It is evidently in the blood itself that salt has its principal function. It is too small in quantity to be very important as an antiseptic, and it is only to its influence on gaseous absorption that we can attribute any important office.

The principal experiments performed upon this point are to be credited to Bernard.

It is estimated that about 20,000 grs. of oxygen may enter the circulation during the twenty-four hours. This amount is subject to great variation. During abstinence much more is absorbed than during digestion.

Salt exercises a direct influence upon this absorption. Bernard placed some blood taken from the jugular vein in two guages. One of these contained pure oxygen, the other oxygen with a weak solution of salt. Upon agitation, the first of these absorbed 20 parts in 100 of the oxygen, the salted blood absorbed 32 parts in 100.

The argument derived from this experiment is, that salt in the blood increases the absorption of oxygen, an argument which has been partially verified by the experiments of Boussingault upon salted forage for cattle. He found that the animals consumed more food with salt than without it; and reasoned thence that it should fatten them. But we only know this. The consumption of salt increases the appetite, and provokes to greater indulgence in food. Under its stimulus a condition stimulating hunger is developed. Now the amount of salt thus taken into the blood is only temporarily there, being soon found in the urine. It is evident that in increasing the absorption of oxygen by the presence of the salt, we must correspondingly increase the conversion of carbon into carbonic acid. Thus though a greater amount of food may be eaten under the stimulus of salt, it does not imply that nutrition is increased, for the waste is also increased.

The office of salt in the blood may be inferred from these experiments and reasonings, viz., to give to the blood a sufficient absorbing power to take in oxygen enough for the maintenance of animal heat.

As animal heat is maintained by the consumption of the carbon of the body, an excess of salt will occasion a too rapid waste of the carbonaceous elements of the system, and consequent emaciation. The dietetical application of this theory is brief, but important. In all those diseases accompanied by waste of the fatty elements, or by rapid pulse and respiration, salt should be withheld. It should not be used by those of thin habit, or with a tendency to tubercular disease.

Those of obese habit should be encouraged in its use. With them it will hasten respiration, quicken the circulation, and increase the waste of the system. In diabetes, which is accompanied by slow respiration and cold surface, (owing to the fact that sugar impedes gaseous absorption,) it will be beneficial by increasing gaseous absorption.

Finally, salt possesses the power of making the red globule of the blood smaller and flatter. In certain malarial diseases the globule is found to be ovoid, and ragged at the edges. In these common salt has been found highly beneficial, and hence antiperiodic powers have been ascribed to it. Inasmnch as it may regulate the circulatory absorption of oxygen, and restore the integrity of the globule, this may be said to be true in a modified and indirect sense.

